# Kinetics of 90° domain wall motions and high frequency mesoscopic dielectric response in strained ferroelectrics: A phase-field simulation

**DOI:** 10.1038/srep05007

**Published:** 2014-05-21

**Authors:** P. Chu, D. P. Chen, Y. L. Wang, Y. L. Xie, Z. B. Yan, J. G. Wan, J.-M. Liu, J. Y. Li

**Affiliations:** 1 Laboratory of Solid State Microstructures, Nanjing University, Nanjing 210093, China; 2 Department of Mechanical Engineering, University of Washington, Seattle, WA 98195, USA

## Abstract

The dielectric and ferroelectric behaviors of a ferroelectric are substantially determined by its domain structure and domain wall dynamics at mesoscopic level. A relationship between the domain walls and high frequency mesoscopic dielectric response is highly appreciated for high frequency applications of ferroelectrics. In this work we investigate the low electric field driven motion of 90°-domain walls and the frequency-domain spectrum of dielectric permittivity in normally strained ferroelectric lattice using the phase-field simulations. It is revealed that, the high-frequency dielectric permittivity is spatially inhomogeneous and reaches the highest value on the 90°-domain walls. A tensile strain favors the parallel domains but suppresses the kinetics of the 90° domain wall motion driven by electric field, while the compressive strain results in the opposite behaviors. The physics underlying the wall motions and thus the dielectric response is associated with the long-range elastic energy. The major contribution to the dielectric response is from the polarization fluctuations on the 90°-domain walls, which are more mobile than those inside the domains. The relevance of the simulated results wth recent experiments is discussed.

Substantial efforts have been devoted to ferroelectric (FE) materials and their applications in advanced electronic technologies^1^,^2^. The core ingredients in the physics of ferroelectrics include electric polarization *P* and its static/dynamic responses to electric field *E_ext_* or/and other stimuli. In the mesoscopic level, the dielectric and polarization responses of a ferroelectric are determined by the specific domain structure and realized by the domain wall motions. In particular, the high-frequency applications of ferroelectrics have received continuous attention. A number of FE materials in thin film form have been used for high-frequency devices in microwave and optical communications as well as computing applications because of their high dielectric permittivity^3^,^4^. A full understanding of the domain wall motion driven by either static or high-frequency dynamic force has been the core issue of the physics of ferroelectrics and the basis for their applications.

The FE domain wall motion is a complicated process and depends on a series of intrinsic particulars such as defects, domain structures, and strains^5^,^6^,^7^. The consequence of strain, a topic to be dealt with in this work, has been highly concerned. Besides conventional scope of ferroelasticity, an externally imposed strain in a paraelectric surprisingly enables remarkable ferroelectricity^8^. Externally imposed strain can modulate the FE phase transitions too^9^,^10^,^11^. These strain effects raise particular attention to FE thin films deposited on rigid substrates, in which the induced strain can be controlled due to the lattice mismatch of the substrates with the films. It thus allows the performance improvement of the FE thin films by means of ‘strain-engineering’ the FE domains in the mesoscopic level.

Along this line, the most interested issue is the strain effect in perovskite FE oxide thin films with tetragonal lattice symmetry like BaTiO_3_ and PbTiO_3_. First, epitaxial BaTiO_3_ and PbTiO_3_ thin films deposited on substrates such as SrTiO_3_, LaAlO_3_, and MgO, are often explored not only for fundamental understanding but also for potential applications, while textured polycrystalline films are also investigated. Second, these thin films offer regular twin-like (stripe-like) 90°-domain structure in coexistence with 180°-domains due to the intrinsic ferroelastic effects. In most cases, the 90° ferroelastic domains are dominant, and the order parameter is strongly coupled to the strains. Furthermore, the substrate induced strain imposes a competitive or coherent coupling with the internal ferroelastic strain in the films, making the domain structure and domain wall motion complicated. These behaviors, particularly in the high frequency range, are much less studied. It is believed that externally imposed strains have strong impacts on the domain wall motion and thus the high frequency dielectric permittivity^12^,^13^,^14^.

A prominent feature of the strain effects is the 90° domain walls vibration in response to an *ac* electric field of high frequency, while the static responses have been well addressed. The dynamics of the domain wall may be characterized by the variation in dielectric permittivity as a function of the *ac* electric field (frequency *ω* and amplitude *E_0_*), as long as the domain structure is well defined. A recent experiment demonstrating this dielectric response was carried out on clamped Pb(Zr_1-*x*_Ti*_x_*)O_3_ (PZT) thin films deposited on Si substrates^15^. By creating cavities beneath the Pt/PZT/Si capacitors and cracking, these released PZT films show dramatic variation in the global dielectric nonlinearity and the frequency dependence as a function of mechanical clamping. The sequent piezoelectric examinations show that the increased local mobility of domain walls must be responsible for these behaviors, where “mobility” refers to the capability of domain walls in response to stimuli. This work among many others raises an important issue on how the 90°-domain structures in FE thin films couple dynamically with externally imposed strains^16^. This question motivates us to investigate the dynamics of domain walls in the 90° domain structure modulated by strains, and consequently the dielectric responses at the mesoccopic level.

While the strain coupling in polycrystalline PZT thin films may be too complicated, our purpose is to focus on tetragonal FE lattice (e.g. BaTiO_3_ or PbTiO_3_) and reveal the dynamics of the 90°-domain structure which couples the internal ferroelastic strains with externally imposed normal strains, in response to electric field *E_ext_*. Instead of addressing the dynamic motion equation^17^,^18^,^19^, we perform the phase-field simulations by which a continuous distribution function of the domain orientations is used to characterize the domain wall motion^20^,^21^. Such a phenomenological method enables us to directly examine the mesoscopic picture of these dynamic processes. We also investigate the underlying mechanism with which the strains modulate the domain wall motion in the energy landscape. In this phase-field model, the dipole-dipole and elastic interactions are considered^22^, since these long-range interactions play an important role in configuring the domain structure^17^,^23^. The strain vector appears as an order parameter and can be modulated by external load.

We consider a tetragonal FE system as approached by an *L* × *L* two-dimensional (2D) lattice with periodic boundary conditions^24^, and start from the Ginzburg-Landau theory. On each site, an electric dipole *P*(*r*)* = *(*P_x_*, *P_y_*) normalized by a pre-factor *P_a_*^25^ and an elastic displacement vector *u*(*r*)* = *(*u_x_*, *u_y_*) are imposed. We clarify that our simulation is not necessarily associated with a thin film, although the electric dipoles in our simulation can only relax on the in-plane configuration. An extension of the results to other geometry is plausible. For simplification, our simulation will not deal with the finite boundary problem since it will introduce complicated corrections to the mechanical balance^25^, while open-circuit boundary issue will only be discussed briefly. Given this assumption, the lattice can be mapped into a small region of real material. By these approximations, we can focus on the details of the local dielectric response in mesoscopic scale.

The electric field *E_ext_* = (*E_x_*, *E_y_*) is confined on the lattice plane. The total free energy for this FE lattice can be written as^26^: 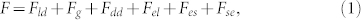
where *F_ld_*, *F_g_*, *F_dd_*, *F_el_*, *F_es_*, and *F_se_* are the Landau potential, gradient energy, dipole-dipole interaction, elastic energy, electrostrictive energy, and electrostatic energy, respectively. Term *F_ld_* extending to the sixth-order is written as: 
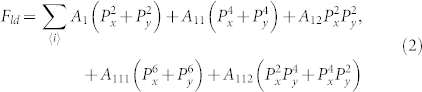
where *A*_1_, *A*_11_, *A*_12_, *A*_111_ and *A*_112_ are the Landau expansion coefficients and *A*_1_ = *A*_10_(*T*-*T*_0_). The lowest-order expression of term *F_g_* is: 
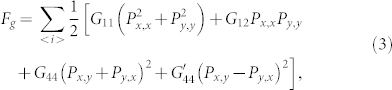
where *P_i,j_ = ∂P_i_/∂r_j_* and *r_j_* = (*x*, *y*), and *G*_11_, *G*_12_, *G*_44_ and *G*′_44_ are the gradient energy coefficients^20^. Terms *F_dd_* and *F_se_* can be written respectively as: 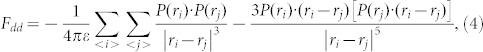

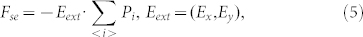
where *F_dd_* includes two contributions. One is the anisotropic part *F_dep_*(*P_0_*) and the other is the isotropic part *F′_dd_*(*δP*) if we treat *P*(*r*) = *P_0_*+*δP*(*r*) where *P_0_* is the spatially homogeneous average polarization and *r* is the spatial vector. It is noted that *P_0_* can be defined by: 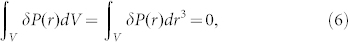
where *V* is the volume of lattice.

Term *F_dep_*(*P_0_*) describes the depolarization energy and shares the same form as *F_se_* by difference of a 1/2 factor. In real system, the depolarization field is compensated by free charges for the cases with open boundaries. For the present case, no free charge is included. The effective electric field is independent of the depolarization field and simply equivalent to *E_ext_*. In experiments, the electric field is usually applied via the electrodes in close-circuit which introduces free charge onto the interface. This will cancel out the depolarization field. Term *F_dd_* counts an integration over the whole lattice, and a realistic calculation is done either by the Fourier transformation or by finite truncation treatment^27^,^28^. For a 2D lattice, the finite truncation is a sufficiently accurate approximation as long as the truncating distance *R* is big (*R* = 8 in our simulation)^24^,^29^,^30^.

The elastic energy *F_el_* yields: 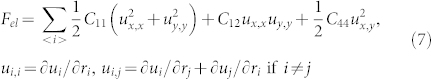
where *C_ij_* is the elastic stiffness tensor which has only three independent elastic constants for a square lattice.

The electrostrictive energy *F_es_* is: 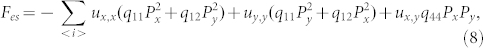
where *q*_11_ = *C*_11_*Q*_11_+2*C*_12_*Q*_12_, *q*_12_ = *C*_11_*Q*_12_+2*C*_12_(*Q*_11_+*Q*_12_), and *q*_44_ = 2*C*_44_*Q*_44_ are the effective electrostrictive coefficients, *Q_ij_* are the electrostrictive coefficients in external stress free state. Mathematically, we can also separate the total strain *η_ij_* into a homogenous component *η_0_*, and a heterogeneous one *δη_ij_* which denotes the microscopic strain distribution at site (*i*, *j*)^22^,^25^: 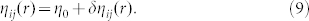
here external strain *η_0_* is imposed either by a substrate or an external load. Similar to generally accepted treatment^20^,^22^,^28^, order parameter *u*(*r*) is reduced to a function of *P*(*r*)^31^. The step-by-step procedure of this derivation can be found in the Supplementary materials. To access the dielectric response over frequency domain, we compute dielectric permittivity *ε*(*ω*) over a broad range of frequency. The algorithms are presented in the Method section. The parameters for the simulations are listed in Table I, and for the details of the simulation procedure one can refer to earlier works^32^,^33^.

## Results

### Domain wall kinetics under dc electric field

We first investigate the 90°-domain wall motion driven by a static (*dc*) *E_ext_*. To characterize the wall motion, we define the domain width *l* deviating from the equilibrium width *l*_0_ under zero electric field. Thus, Δ*l* = *l*-*l_0_* stands for the offset in response to *E_ext_*. In order to minimize the effect of domain wall irregularity, parameters *l* and *l_0_* are calculated indirectly. A set of rectangle-like regions, whose two sides are on the domain walls to enable their areas as big as possible, are taken. We sum the areas of these rectangles. The local domain width *l* is obtained by dividing the area by its length and then performing statistics over sufficient number of rectangles in the same region.

**Figure 1:**
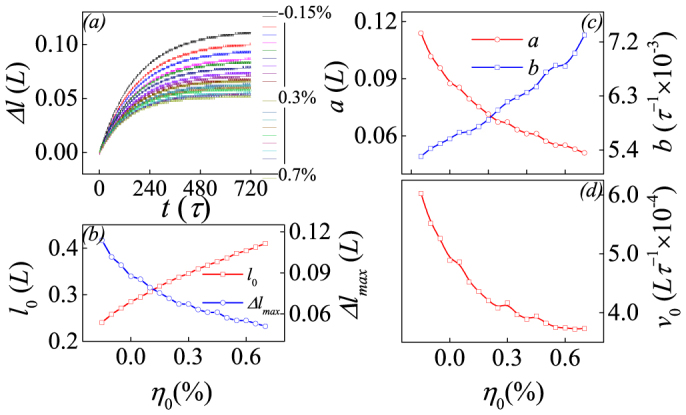
Evaluated parameter Δ*l* as a function of time *t* given a series of *η_0_* labeled numerically (a), *l_0_* and Δ*l_max_* as a function of *η_0_* respectively (b), parameters *a* and *b* as a function of *η_0_* respectively (c), and initial domain wall motion speed *v_0_* as a function of *η_0_* (d). *E_ext_ = *6|A_1_|*P_a_*.

In Figure 1 is shown the kinetics of field-driven domain wall motion, given different strains from slightly compressive ones to tensile ones. Both *E_ext_* and *η_0_* are along the *y*-axis, thus the *l* indicates the width of *a*_2_-domain where the dipoles align along the *y*-axis. The evaluated Δ*l*(*t*) data are plotted in Figure 1(a) where the applied strains are labeled numerically. For each strain, Δ*l*(*t*) shows similar behavior and increases rapidly in the early stage and tends to be saturated at Δ*l_max_* in the late stage. Nevertheless, Δ*l_max_* depends on *η_0_*, and the bigger *η_0_* the smaller Δ*l_max_*. In Figure 1(b) we plot the evaluated *l_0_*(*η_0_*) for the *a*_2_-domain and corresponding Δ*l_max_*(*η_0_*), showing the monotonous increasing of *l_0_*(*η_0_*) and decreasing of Δ*l_max_*(*η_0_*). For definition of each symbol and motion pattern of domain wall, one can refer to the Supplement materials.

The kinetics of wall motion was investigated earlier^34^ and can be described by a simple model^17^,^23^, yielding the kinetic equation Δ*l*(*t*) = *a*(1-*e*^−*bt*^) where *a* = Δ*l_max_* and *b* are the fitting parameters. As shown in Figure 1(c), the monotonous decrease and increase of *a* and *b* as a function of *η_0_* respectively imply that the *a*_2_-domain extension becomes tougher upon the transition from compressive strain to tensile one. It is also possible to evaluate the initial motion speed of the *a*_2_-domain walls by taking *v_0_* = *d*Δ*l*/*dt*|*_t_*_→0_ = *ab*, as shown in Figure 1(d), consistent with the above argument. In other words, for compressive strain (*η_0_*<0), the motion speed of domain walls is higher than that for tensile strain (*η_0_*>0).

For the cases with strain *η_0_* not parallel to *E_ext_*, the above analysis is qualitatively correct. The tensile strain makes the domains with *P*//*η_0_* wider and the compressive one makes it narrower. This behavior allows a competition between the strain effect and electric field effect, and they can be coherent or cancelled depending on their orientation relationship. However, when the strain contains shear components, the situation becomes more complicated and extensive calculation is not discussed here.

### Domain wall vibrations and dielectric response: η_0_ = 0

Based on the above result, one understands that the 90°-domain structure clamped by external stress has different stability characteristics from the stress-free state. This difference can be discussed in the clamped 90°-domain structure driven by the *ac*-electric field, characterized by variation of dielectric permittivity as a function of *η_0_*. In particular, the dielectric response in the frequency domain is related to the wall motion.

**Figure 2:**
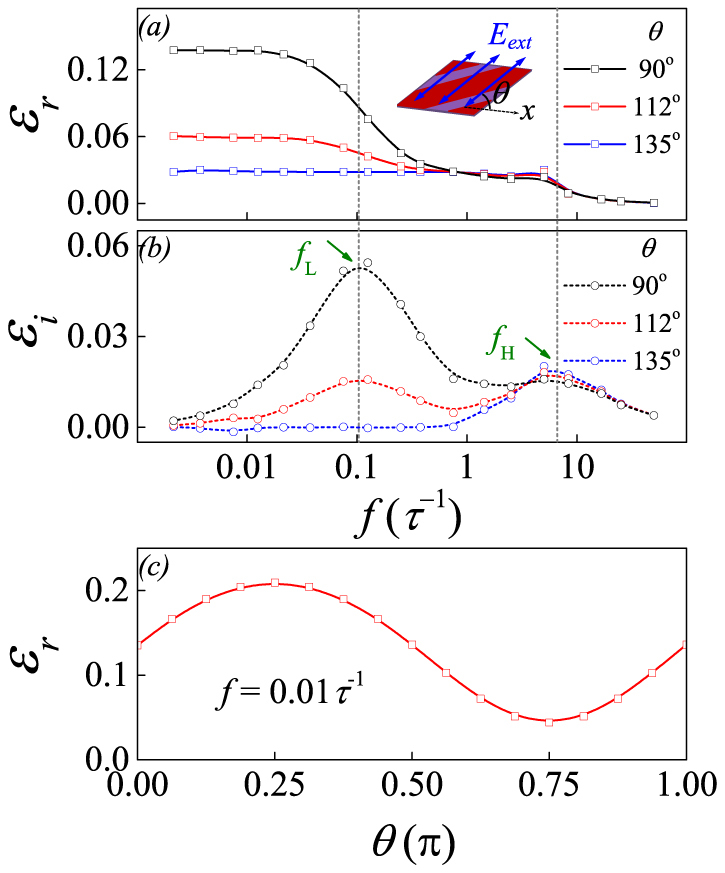
Simulated dielectric permittivity spectra: (a) real part and (b) imaginary part over a wide range of frequency, at *θ* = 90°, 112°, and 135°, respectively. (c) Dielectric permittivity real part *ε_r_* as a function of angle *θ* at a constant *f* = 0.01*τ*^−1^.

We first address the dielectric response in *η_0_* = 0. Figure 2(a) and (b) show the real part *ε_r_*(*f*) and imaginary part *ε_i_*(*f*) at three *θ* angles. Here a small *E_0_* = 0.6|A_1_|*P_a_* is chosen, with the field direction defined by angle *θ* between the *x*-axis and *E_ext_*. In general, *ε_r_*(*f*) decreases gradually with increasing *f* for all the three cases, while *ε_i_*(*f*) shows two peaks at characteristic frequencies *f_L_* ~ 0.1*τ*^−1^ and *f_H_* ~ 7*τ*^−1^, which are respectively referred as the low-*f* and high-*f* dispersions. Here *τ* = 1/|*A*_1_|*D* is the characteristic time for electric dipole flip (see the Method section for details). The striking feature is the low-*f* dispersion anisotropy, i.e. the *θ*-dependence which is the most remarkable at *θ* = 90° (and *θ* = 0 too, four-fold symmetry). This *θ*-dependence is shown by *ε_r_*(*θ*) at *f* = 0.01*τ*^−1^ as an example in Figure 2(c), characterized by the typical periodic variation.

The above behaviors can be understood by investigating the instant evolution of domain structure. By turning *E_ext_* from *θ* = 0 to *θ* = 180°, one checks the domain evolution and dielectric dispersion. The low-*f* dispersion is related to the domain wall vibrations, while the high-*f* dispersion is attributed to the flip of individual dipoles. In fact, the dispersion peak around *f_H_* is also observed in the mono-domain lattice.

**Figure 3:**
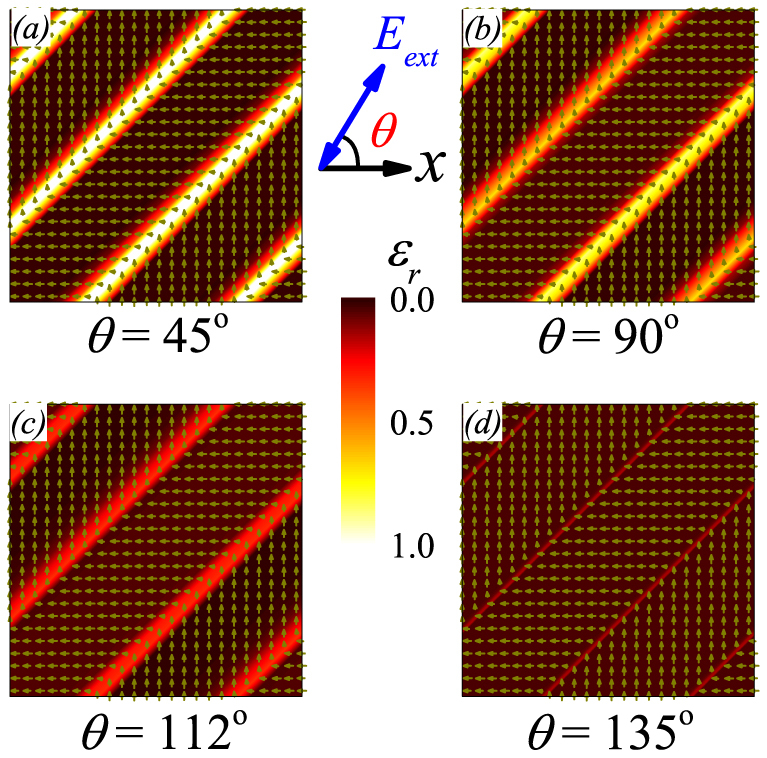
Snapshoted patterns of dielectric permittivity real part *ε_r_* at constant frequency *f* = 0.05*τ*^−1^ with angle *θ* = 45° (a), 90° (b), 112° (c), and 135° (d).

Now we investigate the origin for the low-*f* dispersion anisotropy in the domain scale. In *η_0_* = 0, the spatial distributions of *ε_r_* at *f* = 0.05*τ*^−1^ in four *θ* angles are presented in Figure 3(a)~(d), respectively, where the color scales the intensity (*ε_r_* = 0.0~1.0). It is immediately seen that the dielectric permittivity mainly comes from the contribution of wall vibrations, while those dipoles deeply inside the domains contribute little. This characteristic makes the dielectric response very specific.

At *θ* = 45°, the domain structure and dielectric response are shown in Figure 3(a). Again, the dielectric response mainly comes from the contribution of electric dipoles on the walls and near regions. This feature can be understood from the electrostatic energy -*P***·***E_ext_*. The walls have the highest local mobility. No matter *E_ext_* is positive or negative, there is always one of the neighboring two domains, inside which all the dipoles take the 135° angle from *E_ext_*. This domain will shrink while the other will expand, making the wall move easily. This is also the reason why *ε_r_* is the highest at *θ* = 45°, as seen in Figure 2(c).

At *θ* = 90° (or *θ* = 0), as shown in Figure 3(b), the wall mobility is slightly lower, and *ε_r_* is lower too. In this case, *E_ext_* is parallel to the dipoles in one domain and perpendicular to those in the other. At *θ* = 112° and *θ* = 135°, as shown in Figure 3(c) and (d), respectively, the wall mobility falls down even further, resulting in even lower *ε_r_*. The wall mobility becomes the lowest at *θ* = 135°. Figure 3(d) shows that almost the whole lattice has the identical *ε_r_* except the very thin and dim lines on the walls. The reason is that term -*P***·***E_ext_* in two neighboring domains are almost equivalent and they compete with each other, hindering the wall motion. The dielectric response is weak with no frequency dispersion.

**Figure 4:**
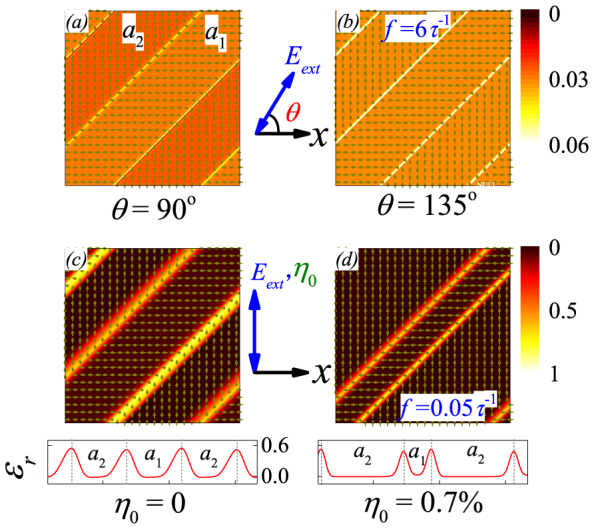
Snapshoted patterns of dielectric permittivity real part *ε_r_* at constant frequency *f* = 6*τ*^−1^ with angle *θ* = 90° (a) and 135° (b). At constant frequency *f* = 0.05*τ*^−1^ with different tensile strains along the *y-*axis: (c) *η_0_* = 0 and (d) *η_0_* = 0.7%. The red lines below the snapshots indicate the alignment of *ε_r_* along the 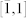. Hereafter, *E_0_ = *0.6|A_1_|*P_a_*.

We finally check the dielectric response in the high-*f* range and one example is given in Figure 4(a) and (b) at *f* = 6*τ*^−1^ for two specific *θ* angles: *θ* = 90° and *θ* = 135°. The dielectric distribution over the whole lattice is roughly homogeneous. For details, one looks at the case in Figure 4(a) and finds only weak color contrast between the two neighboring domains *a*_1_ and *a*_2_. The domain *a*_1_ whose dipoles align along the *x*-axis but perpendicular to *E_ext_* shows slightly higher *ε_r_* than that of domain *a*_2_. The reason is obvious that the dipoles in domain *a*_1_ are more fluctuating than those in domain *a*_2_.

### Domain wall vibrations and dielectric response: η_0_>0

**Figure 5:**
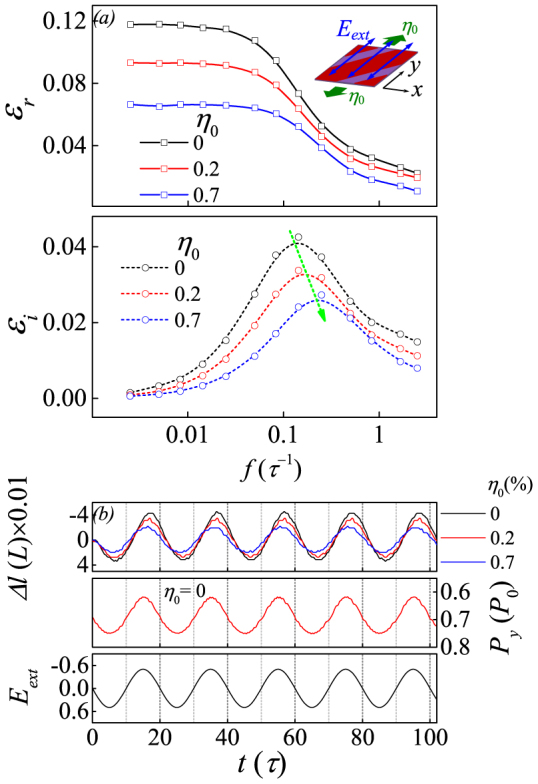
(a) Simulated dielectric permittivity spectrum around *f* = 0.05*τ*^−1^ given *η_0_* = 0, 0.2%, and 0.7% along the *y*-axis. (b) From top to bottom: parameter Δ*l* as a function of time *t* given *η_0_* = 0, 0.2%, and 0.7% at *f* = 0.05*τ*^−1^, the *y* component of total polarization as a function of time *t*, and the *ac* electric field *E_ext_* as a function of time *t*. The *ac* electric filed is along *y*-axis, *E_0_ = *0.6|A_1_|*P_a_*.

Now we discuss the cases with *η_0_*≠0. We only present in details the results on normally strained lattice. When the lattice is normally strained, the domain structure is deformed. To clarify the similarity and difference between the strain-free and strained lattices, we consider the simplest situation: *η_0_* = 0.7% under *E_ext_* with *f* = 0.05*τ*^−1^ and *E_0_* = 0.6|A_1_|*P_a_*, both aligned along the *y*-axis (*θ* = 90°). We present in Figure 4(c) and (d) the strain-free domain structure and strained structure respectively, as well as the spatial distribution of *ε_r_*. For the two cases, the distributions are similar in amplitude but the high-*ε_r_* spatial profiles across the domain walls are much wider for the strain-free lattice than those for the strained lattice. In the qualitative sense, the dielectric permittivity averaged over the whole strained lattice is lower than that over the strain-free lattice, at least in the low-*f* range. This *η_0_*-dependence can be further illustrated in Figure 5(a), where *ε_r_*(*f*) and *ε_i_*(*f*) at three tensile strains (*η_0_* = 0, 0.2%, 0.7%) are plotted. Both the real and imaginary parts are remarkably suppressed by tensile strain, and the *f_L_* has a slight shift towards the high-*f* side. We also calculate the instant responses of the Δ*l* and polarization component *P_y_* against *E_ext_* at the three tensile strains, shown in Figure 5(b). The one-to-one correspondence between Δ*l*, *P_y_*, and *E_ext_*, respectively, is observed. The responses of Δ*l* and *P_y_* are synchronous with *E_ext_* in the low-*f* range, and delayed in the high-*f* range. Furthermore, the wall vibration amplitude is suppressed by the tensile strain.

The above results refer to the simplest situation. When *η_0_* and *E_ext_* are aligned along arbitrary directions independently, more complexity is seen in terms of the domain structure evolution and dielectric response. For some specific geometry, the strain and electric field may compete with each other. Some more discussion will be given below. However, in general, the calculated results are qualitatively similar to those for the simplest situation: the tensile strain suppresses the domain wall vibration, thus reducing the dielectric permittivity.

### Domain wall vibrations and dielectric response: η_0_<0

**Figure 6:**
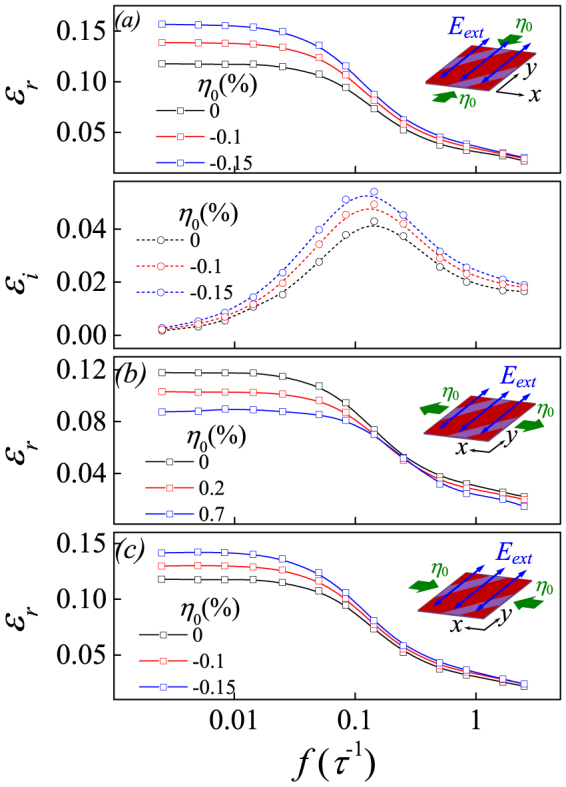
(a) Simulated dielectric permittivity spectrum around *f* = 0.05*τ*^−1^ given *η_0_* = 0, −0.1%, and −0.15% along the *y*-axis with the *ac* electric along the *y*-axis too. The calculated real part *ε_r_* at tensile strain (b) and compressive strain (c) perpendicular to the *ac* electric field along the *y*-axis. *E_0_ = *0.6|A_1_|*P_a_*.

Now we check the cases with *η_0_*<0. Referring to the results under static (*dc*) *E_ext_*, as shown in Figure 1, one sees that the static compressive strain assists the 90° domain wall motion. It is thus expected that the dielectric permittivity in compressed lattice will increase. The *η_0_*-dependences of *ε_r_*(*f*) in three compressive strains (*η_0_* = 0, −0.1%, −0.15%) along the *y*-axis are plotted in Figure 6(a), consistent with the expected results. Similar behaviors are observed for the strain and electric field both applied along the *x*-axis.

**Figure 7:**
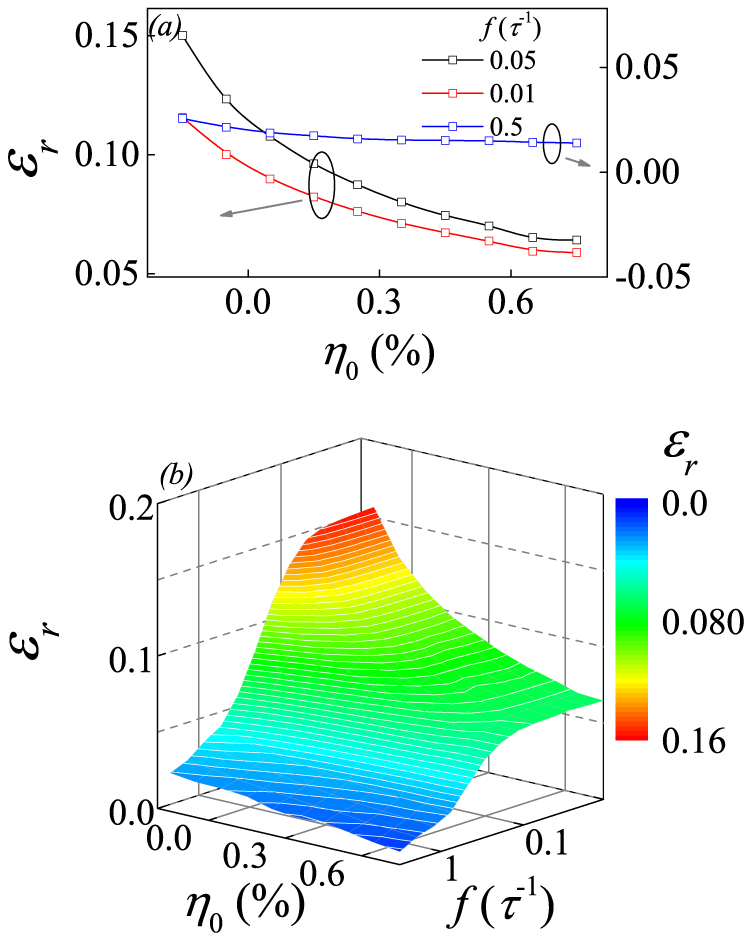
(a) Simulated dielectric permittivity real part *ε_r_* as a function of *η_0_* with the *ac* electric field frequency *f* = 0.01*τ*^−1^, 0.05*τ*^−1^, and 0.5*τ*^−1^. (b) A 2D plot of *ε_r_* as a function of *η_0_* and *f*. The *ac* electric field and strains are all along the *y*-axis. *E_0_ = *0.6|A_1_|*P_a_*.

We also check the situations where *E_ext_* is not parallel to *η_0_*. One example is shown in Figure 6(b) for *η_0_*>0 and Figure 6(c) for *η_0_*<0, where *E_ext_* is along the *y*-axis and *η_0_* is along the *x*-axis. Our extensive calculations establish the qualitatively similar behaviors: the tensile strain suppresses the 90° domain wall motion and thus the dielectric permittivity, while the compressive strain enhances the domain wall motion and the dielectric permittivity, no matter whether *E_ext_* is not parallel to *η_0_* or not. We summarize spectrum *ε_r_*(*f*, *η_0_*) in Figure 7(a) and (b). At *f*<*f_L_*, such as *f* = 0.01*τ*^−1^ and 0.05*τ*^−1^, *ε_r_* falls gradually with increasing *η_0_* from *η_0_*<0 to *η_0_*>0, while this tendency becomes negligible as *f*>*f_L_* such as *f* = 0.5*τ*^−1^ since the single dipole response becomes dominant at this frequency. The overall evolution of *ε_r_*(*f*, *η_0_*) is plotted in Figure 7(b).

## Discussion

It should be mentioned that the periodic boundary conditions allows the total strains in all directions to be exactly balanced out, and the strain effect is of long-range and coupled with mechanical boundary conditions. However, if other mechanical boundary conditions are considered, such as free boundary, the total strains can be partially relaxed through the free boundaries. The 90°-domain walls can be more moveable and thus show more significant response to electric field, as partially discussed in Ref. 15.

To understand the simulated results, one may give additional discussion by looking at the energy landscape and comparing the simulated results and experiments, although relevant experimental data are really rare.

### Energy landscape

We calculate the elastic energy distribution associated with the 90° domain structures. For a single domain lattice, the total strain field is homogeneous, thus accommodating extremely large elastic energy. The single domain is decomposed into the 90° domain structure so that the total elastic energy can be relaxed. Owing to the lattice volume conservation, for one domain, the compressive strain along one direction (e.g. the *x*-axis) is always accompanied with the tensile strain along the other direction (the *y*-axis), and verse vice. One can refer to the Supplement materials for the strain distribution.

**Figure 8:**
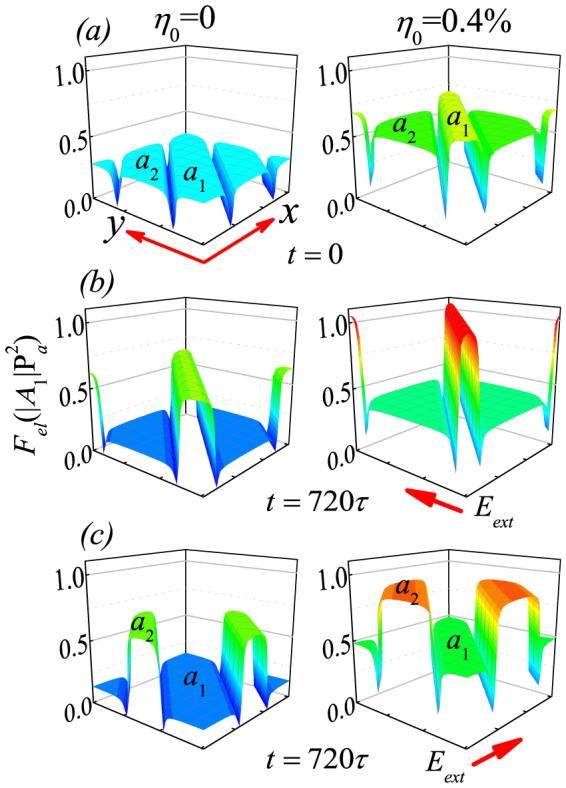
Evaluated spatial contours of elastic energy *F_el_* in the 90°-domained lattice. The left column (a) refers to *η_0_* = 0 and the right one (b) refers to *η_0_* = 0.4%. The top row refers to *E_ext_* = 0, the middle row refers to *t* = 720*τ* after *E_ext_* (*dc*) along the *y*-axis applies to the lattice, and the bottom row refers to *t* = 720*τ* given *E_ext_* (*dc*) along the *x*-axis. *E_ext_ = *6|A_1_|*P_a_*.

First, we consider the *η_0_* = 0 case. Given a static electric field along the *y*-axis, the domain walls move into the *a*_1_-domain in compensation with the extension of the *a*_2_-domain width. The continuous shrinking of the *a*_1_-domain is accompanied with the increasing magnitude of elastic strain (*e_xx_* and *e_yy_*) inside the *a*_1_-domain. Since the total elastic energy is proportional to *η*^2^, the rapidly enhanced total elastic energy inside the shrunk *a*_1_-domain acts as the resistant force against the electric field, responsible for eventual termination of the wall motion when the *a*_1_-domain becomes sufficiently narrow, as shown in the left column of Figure 8(a) and Figure 8(b), where the total elastic energy *F_el_*(*x*, *y*) is plotted. This explains why *Δl* tends to be saturated at *Δl_max_* as time goes infinitive.

This scenario applies to the lattice with externally imposed strain. Take *η_0_* = 0.4% along the *y*-axis as an example. At *t* = 0 with *E_ext_* = 0, the whole elastic energy distribution on the right column of Figure 8 shifts upward with respect to the case of *η_0_* = 0. However, one can observe a much higher energy distribution in the shrinking *a*_1_-domains. Therefore, the *a*_1_-domains are already under highly tensile state along the *x*-axis even at *E_ext_* = 0, when an externally imposed strain along the *y*-axis is applied. In this case, a static electric field along the *y*-axis may further force the wall moving into the *a*_1_-domain but will be highly resisted by the high strain energy, as shown in the right column of Figure 8. Therefore, additional shrinking of the *a*_1_-domain driven by the electric field would become even tougher, given that *η_0_* = 0.4% already makes the *a*_1_-domain narrow.

If strain *η_0_* is applied along other orientations, no matter whether it is parallel to *E_ext_* or not, the wall motion behaviors remain qualitatively similar. One example is given by applying *E_ext_* along the *x*-axis where *E_ext_* and *η_0_* are normal to each other. As shown in Figure 8(c), a strain *η_0_* along the *y*-axis enhances remarkably the total elastic energy in the *a*_2_-domain, making the further shrinking of the *a*_2_-domain more difficult than the case with *η_0_* = 0. Therefore, the above-discussed results don't lose the generality.

### Comparison with experiments

The implication of the above simulation data can be checked by comparing the simulated results with experimental data on the contribution of domain wall vibrations to the dielectric response in BaTiO_3_ (BTO) and Pb(Zr_1-x_Ti_x_)O_3_ (PZT) ceramics^35^,^36^,^37^, although these data have not yet received confirm from the direct detecting of the mesoscopic scale dielectric distribution across the 90° domain walls. Therefore, such comparisons may not be so direct in quantitative sense. For PZT, experiments^35^ showed that above 10^11^ Hz the real part of dielectric constant begins to drop, due to the frozen dipole oscillators themselves. In our simulation, the corresponding frequency is assigned as *f_H_* = 7*τ*^−1^ in Figure 2(a). Thus we have the characteristic inverse time scale *τ*^−1^ ~10^10^ s^−1^, and the calculated *f_L_* ~10^9^ Hz.

Regarding the PZT ceramics, experimental data^36^ also indicate the dielectric dispersion around 1.0 GHz, which was argued to arise from the vibrations of the frozen 90° domain walls. For the dielectric permittivity magnitude, we take the dimensionless factor (|*A*_1_|ε*)^−1^~10^3^, where *A*_1_ is 3.8(*T*-479) × 10^5^C^−2^m^2^N taken from PZT and ε* is the vacuum permittivity. The calculated real part of the dielectric permittivity at *θ* = 45° is ~200, as shown in Figure 2(c), and the maximum of the imaginary part is ~100. Indeed, experimental measurements^37^ on ceramics PZT (*x* = 0.48–0.52) gave the difference in the real part between the extremely high frequency and the very low frequency, which is 250–500. The peak of the imaginary part is 150–265. These values agree roughly with our simulation results here. The above comparisons allow us to claim that the present calculations are reliable even in quantitative sense.

We also find some experimental results about the effects of tensile and compressive strains on the overall dielectric constant. In microwave frequency range, it was shown that dielectric constant and tunability of BTO films grown on MgO gradually decrease as the in-plane strain goes from the compressive type to tensile type^38^. Similar behaviors were observed in polycrystalline Ba_0.6_Sr_0.4_TiO_3_ thin films upon increasing tensile strain^39^. Those results are consistent with our calculation in a qualitative sense. Unfortunately, none of those experiments establishes a clear logic between the dielectric response and the 90° domain wall motion, although the frequency range is properly related to the 90° domain wall vibration. Establishing this logic is a technical challenge for experimentalists. To reveal the domain wall response to external electric field, one may map the local polarization response in real-space. Nevertheless, this task becomes difficult for such a high frequency. In this sense, the present simulation seems to be unique for bridging the relationship between the dielectric response in microwave frequency and the strain via the microscopic domain scale.

## Methods

### Calculation of *ac* dielectric permittivity

The temporal evolution of the dipole lattice is tracked by solving the Ginzburg-Landau (TDGL) equation which takes the following form: 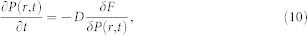
where *t* is time scaled in unit *τ* with *τ* ^−1^ = |*A*_1_|*D*, and *D* is the kinetic coefficient. We also calculate the dielectric permittivity as a function of *E_ext_*. For a dielectric system that cannot polarize instantaneously in response to an electric field, the total electric polarization as a function of *t* can be described as: 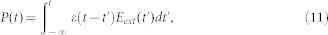
where *P*(*t*) is a convolution of electric field *E*(*t*) at previous times with time-dependent permittivity *ε*(*t*) = *ε_r_*(*t*)+*iε_i_*(*t*). Therefore, its Fourier transformation can be directly written as 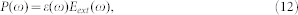
where *P*(*ω*) and *E_ext_*(*ω*) are the Fourier transformations of *P*(*t*) and *E_ext_*(*t*) respectively^32^. For the case of *ac* sine electric field such as *E_ext_*(*t*)* = E_0_***·**sin(*ω*_0_*t*) with *ω*_0_ = 2π*f_0_* and *ω* = 2π*f*, *E_ext_*(*ω*) can be directly written as: 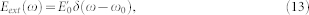
where *E*′_0_ is the coefficient of Fourier transformation and *ω*_0_ is the frequency. Polarization *P*(*ω*) can be calculated by Fourier-transforming the temporal evolution spectrum *P*(*r*, *t*) in Eq.(10). In details, the real-spatial spectrum of *P*(*r*, *t*) at site *r* and its spatial average *P*(*t*) over sufficient number of time periods is calculated by solving Eq.(10). The Fourier-transformed frequency spectrum *P*(*r*, *ω*) can be expressed as: 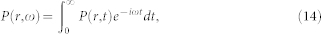
which is used to transform *P*(*r*, *t*) and *P*(*t*) into frequency domain to obtain *P*(*r*, *ω*) and *P*(*ω*). Subsequently, one can compute the corresponding dielectric permittivity *ε*(*r, ω*_0_) at site *r* and its spatial average *ε*(*ω*_0_) at frequency *ω*_0_ using Eq.(12).

## Author Contributions

J.M.L. conceived the research project and P.C. performed the computations. D.P.C., Y.L.W., Y.L.X., Z.B.Y., J.G.W. and J.Y.L. commented the modeling and discussed the results. P.C. and J.M.L. wrote the paper.

## Supplementary Material

Supplementary Information Supplementary Materials

## Figures and Tables

**Table 1:** Physical parameters chosen for the simulation (*τ* ^−1^ = |*A*_1_|*D*) [24, 25]. All these parameters appear in the dimensionless form

Parameter (unit)	Value	Parameter (unit)	Value	Parameter (unit)	Value
*L*	64~256	A*_1_ (|*A*_1_|)	−1.00	A*_11_ (*A*_11_*P*^2^_0_/|*A*_1_|)	−0.24
A*_12_ (*A*_12_*P*^2^_0_/|*A*_1_|)	2.50	A*_111_ (*A*_111_*P*^4^_0_/|*A*_1_|)	0.49	A*_112_ (*A*_112_*P*^4^_0_/|*A*_1_|)	1.20
G*_11_ (*G*_11_/*A*^2^_1_|*A*_1_|)	1.60	G*_12_ (*G*_12_/*A*^2^_1_|*A*_1_|)	0.00	G*_44_ (*G*_44_/*A*^2^_1_|*A*_1_|)	0.80
*G*′*_44_ (*G′*_44_/*A*^2^_1_|*A*_1_|)	0.80	C*_11_ (*C*_11_/|*A*_1_|*P*^2^_0_)	2.75	C*_12_ (*C*_12_/|*A*_1_|*P*^2^_0_)	1.79
C*_44_ (*C*_44_/|*A*_1_|*P*^2^_0_)	0.543	q*_11_ (*q*_11_/|*A*_1_|)	0.143	q*_12_ (*q*_12_/|*A*_1_|)	−0.0074
q*_44_ (*q*_44_/|*A*_1_|)	0.0157	τ* (*τ*)	0.0004		
