# Supplementary material for: Kinetics of 90° domain wall motions and high frequency mesoscopic dielectric response in strained ferroelectrics: A phase-field simulation
Source: Sci Rep. 2014 May 21;4:5007. doi: 10.1038/srep05007 (PMC4028899; doi:10.1038/srep05007)
Supplement: Supplementary Information — Supplementary Materials [file srep05007-s1.pdf]

Supplementary materials to Scientific Reports (Ref. SREP-14-01932)

Kinetics of 90° domain wall motions and high frequency mesoscopic dielectric response in strained ferroelectrics: A phase-field simulation

P. Chu[1], D. P. Chen[1], Y. L. Wang[1], Y. L. Xie[1], Z. B. Yan[1], J. G. Wan[1], J. –M. Liu[1] *, and J. Y. Li[2]

[1]Laboratory of Solid State Microstructures, Nanjing University, Nanjing 210093, China

[2]Department of Mechanical Engineering, University of Washington, Seattle, WA 98195, USA

*Correspondence and requests for materials should be addressed to J. –M. Liu (email: liujm@nju.edu.cn).

1. Elastic strain energy of a domain structure

In the present framework, the 90°-domain walls and their dynamics are associated with the total strain which includes the ferroelastic strain and externally imposed normal strain. The ferroelastic strain associated with the FE polarization field is given by:

$$\begin{aligned}\eta_{11}^0 &= Q_{11}P_x^2 + Q_{12}P_y^2 \\ \eta_{22}^0 &= Q_{11}P_y^2 + Q_{12}P_x^2 , \\ \eta_{12}^0 &= Q_{44}P_xP_y\end{aligned} \quad \text{(S1)}$$

where Q_{ij} are the electrostrictive coefficients in the external stress free state.

The elastic strain e_{ij} and the related elastic energy F_e can be expressed as:

$$\begin{aligned}&e_{ij} = \eta_{ij} - \eta_{ij}^0, \quad (i, j = 1, 2) \\ &F_e = \frac{1}{2}\sum_{<ijkl>} c_{ijkl}e_{ij}e_{kl} = \frac{1}{2}\sum_{<ij>} c_{ijkl}\left(\eta_{ij} - \eta_{ij}^0\right)\left(\eta_{kl} - \eta_{kl}^0\right), \quad (k, l = 1, 2)\text{'}\end{aligned} \quad \text{(S2)}$$

where η_{ij} is the total strain, c_{ijkl} is the elastic stiffness tensor which has only three independent elastic constants C_{11}, C_{12}, and C_{44} for a square lattice. Combining Eq.(S1) with Eq.(S2) gives term $F_e = F_{el} + F_{es} + F'_e$, i.e. term F_e can be decomposed into three terms F_{el}, F_{es}, and F'_e. Term F'_e is a function of polarization $P(r)$ and thus only affects the Landau expression, i.e. A_{11} and A_{12}.

In our simulation the order parameter $u(r)$ is reduced to a function of $P(r)$ based on the assumption that the response time of elastic lattice strain is far shorter than the time for electric dipole relaxation. In that view, we can use a static condition for mechanical equilibrium to solve the displacement field $u(x, y)$ as a function of the polarization field $P(x, y)$. The mechanical equilibrium equation is expressed as $\sigma_{ij,j}=0$, and stress tensor σ_{ij} is related to strain e_{ij} according to the Hooke's law via $\sigma_{ij}=c_{ijkl}e_{kl}=c_{ijkl}(\eta_{kl}-\eta^{0}_{kl})$. Therefore, the equilibrium equation can be rewritten as:

$$c_{ijkl}u_{k,lj} = c_{ijkl}\eta^{0}_{ki,j}\,, \qquad \text{(S3)}$$

which has a solution in the Fourier space, given a periodic boundary conditions:

$$\hat{u}_i = -\sqrt{-1}g_{ij}c_{jmkl}\hat{\eta}^{0}_{kl}\zeta_m,\ \ (m=1,2)\,, \qquad \text{(S4)}$$

where $g_{ij}^{-1}=c_{ijkl}\zeta_k\zeta_l$ and ζ is the coordinate in the Fourier space, $\hat{u}_i$ and $\hat{\eta}^{0}_{kl}$ are the Fourier transform of u_i and η^{0}_{ij}, respectively. We can use the inverse Fourier transformation to obtain u_i in real space.

2. Domain wall motion under *dc* electric field

Figure S1 present two columns of snapshoted images of the domain structure at various times after a static electric field $E_{ext}=6|A_1|P_a$ is applied along the y-axis at t=0. The left column (a) refers to the case of external strain η_0=0 and the right one (b) to the case of η_0=0.4% (tensile strain) along the y-axis too (i.e. $\eta_0//E_{ext}$). The polarization in each domain is indicated by the black arrows and the a_1-domain stands for $P//x$-axis and a_2-domain for $P//y$-axis. Parameter l_0 and Δl are labeled for guide of eyes. By comparing the domain patterns in the two columns, it is clearly seen that the tensile strain along the y-axis not only broadens the domains with polarization parallel to the strain direction (a_2-domains) but also remarkably delays the domain wall motion, i.e. the local mobility of the domain walls is reduced. Although the a_2-domains for η_0=0.4% are eventually wider than those for η_0=0, which is the strain induced effect, the Δl value, induced by E_{ext}, is much smaller in this case. The domain wall motion will be eventually terminated, approaching another equilibrium state, as shown by the images at $t=720\tau$.

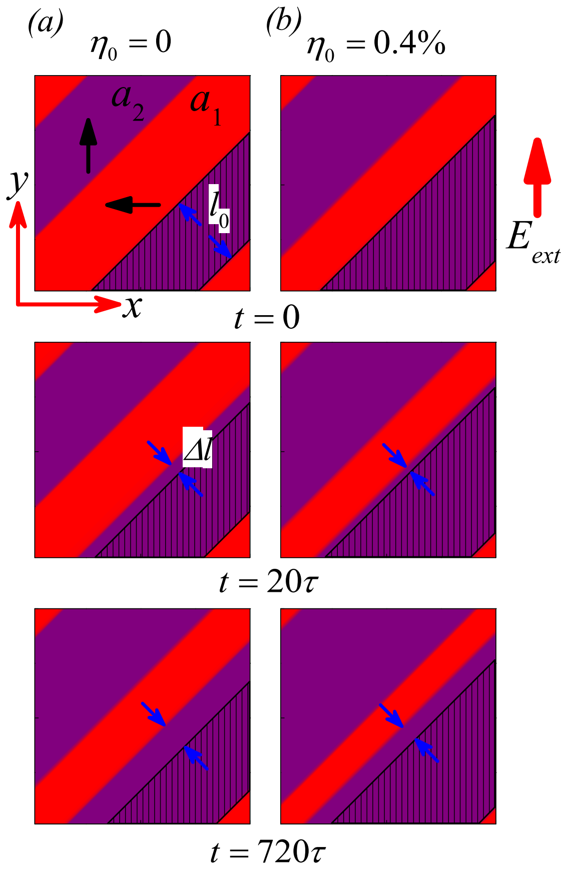

Figure S1. (color online) Simulated 90°-domain structures. The left column (a) refers to η_0=0 and the right one (b) refers to η_0=0.4%. The top row refers to E_{ext}=0, and the middle and bottom rows refer to t=20τ and t=720τ after field E_{ext} is applied to the lattice. $E_{ext}=6|A_1|P_a$.

3. Evolution of underlying strain distribution

Figure S2 evaluates the spatial contours of strain e_{xx} and e_{yy} in each step of Figure S1. The red surfaces stand for e_{xx} and blue ones for e_{yy}. Owing to the lattice volume conservation, for one domain, the compressive strain along one direction (e.g. the x-axis) is always accompanied with the tensile strain along the other direction (the y-axis), and verse vice. As shown in Fig.S2(a) at t=0, the a_1-domain accommodates the tensile and compressive strains along the x-axis and y-axis respectively, while the compressive and tensile strains along the x-axis and y-axis are developed in the a_2-domain.

First, we consider the η_0=0 case. Given a static electric field along the y-axis, the domain walls move into the a_1-domain in compensation with the extension of the a_2-domain width, and the electric field serves as the driving force of the wall motion. The continuous shrinking

of the a_1-domain is accompanied with the increasing magnitude of both e_{xx} and e_{yy} inside the a_1-domain (e_{xx} from 0.2% to 0.5% and e_{yy} from -0.5% to -0.8%) with time. Correspondingly, both e_{xx} and e_{yy} inside the a_2-domain have their magnitudes decreasing with time (e_{xx} from -0.5% to -0.3% and e_{yy} from 0.2% to 0).

When externally imposed strain, the calculated spatial distributions of e_{xx} and e_{yy} in the a_1-domain and its neighbor a_2-domain are shown in the Fig.S2(b). At t=0 with E_{ext}=0, the whole strain field contour shifts upward with respect to the case of η_0=0. However, a careful observation shows that both the tensile strain e_{xx} in the a_1-domain and tensile strain e_{yy} in the a_2-domain are increased by ~0.3%, while the reductions of the compressive e_{yy} in the a_1-domain and compressive e_{xx} in the a_2-domain are much less (~0.1%). Therefore, additional shrinking of the a_1-domain driven by the electric field would become even tougher, given that η_0=0.4% already makes the a_1-domain narrow.

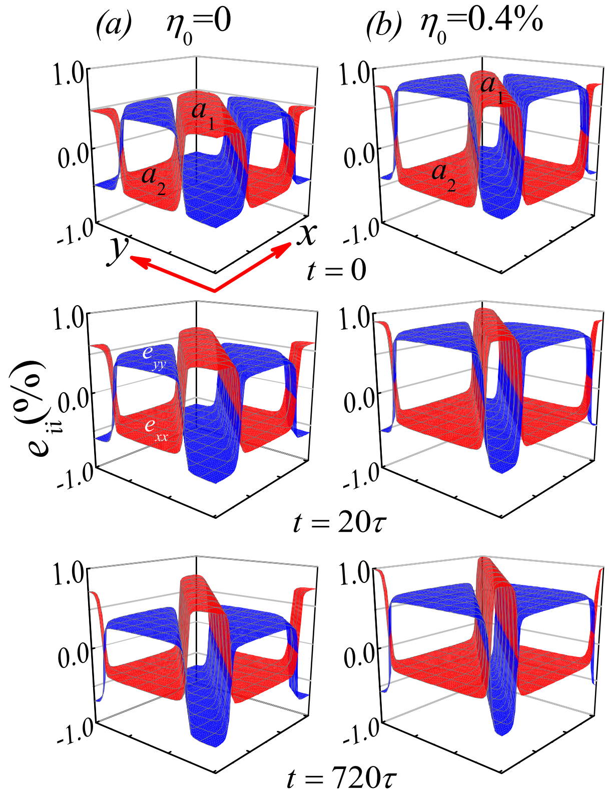

Figure S2. (color online) Evaluated spatial contours of strain e_{xx} and e_{yy} in the 90°-domained lattice. The red surfaces stand for e_{xx} and blue ones for e_{yy}. The left column (a) refers to η_0=0 and the right one (b) refers to η_0=0.4%. The top row refers to E_{ext}=0, and the middle and bottom rows refer to t=20τ and t=720τ given E_{ext} (dc) along the x-axis. E_{ext}=0.6$|A_1|P_a$.
